# Development and expansion in the marine social sciences: Insights from the global community

**DOI:** 10.1016/j.isci.2022.104735

**Published:** 2022-07-11

**Authors:** Emma McKinley, Rachel Kelly, Mary Mackay, Rebecca Shellock, Christopher Cvitanovic, Ingrid van Putten

**Affiliations:** 1 School of Earth and Environmental Sciences, Cardiff University, Park Place, Cardiff, CF10 3AT, UK; 2 Centre for Marine Socioecology, University of Tasmania, Hobart, TAS 7005, Australia; 3 Future Ocean and Coastal Infrastructure Consortium, Memorial University of Newfoundland, St. John’s, NL A1C 5S7, Canada; 4 CSIRO Oceans and Atmosphere, Castray Esplanade, Hobart, TAS 7000, Australia; 5 Australian National Centre for the Public Awareness of Science, Australian National University, Canberra, Australia

**Keywords:** Earth sciences, Marine processes, Social sciences, Research methodology social sciences

## Abstract

The importance of understanding the complexities of societal relationships with our global ocean, and how these influence sustainable management and effective, equitable governance, is crucial to addressing ocean challenges. Using established horizon scanning method, this paper explores current trends in marine social sciences through a survey of the global marine social science research and practitioner community (n = 106). We find that marine social sciences research is broad, covering themes relating to governance and decision-making, stakeholder participation and engagement, the socio-cultural dimensions of marine systems, ocean literacy, community-based and area-specific management, and the blue economy, and identify future research priorities highlighted by the community. Our results, however, suggest several barriers persist, including the relationship between marine social sciences and other disciplines, and the visibility and recognition of marine social sciences both internal and external to academia. Finally, the paper generates prospective thinking and highlights recommendations for future research and practice.

## Highlights

• Presents the results of a global marine social science horizon scan (n = 106)
• Future priorities include blue economy, social justice, and ocean literacy
• Reputation, funding, and capacity building remain key challenges
• Enablers are presented to overcome barriers to marine social science research

## Graphical abstract

**Figure:**
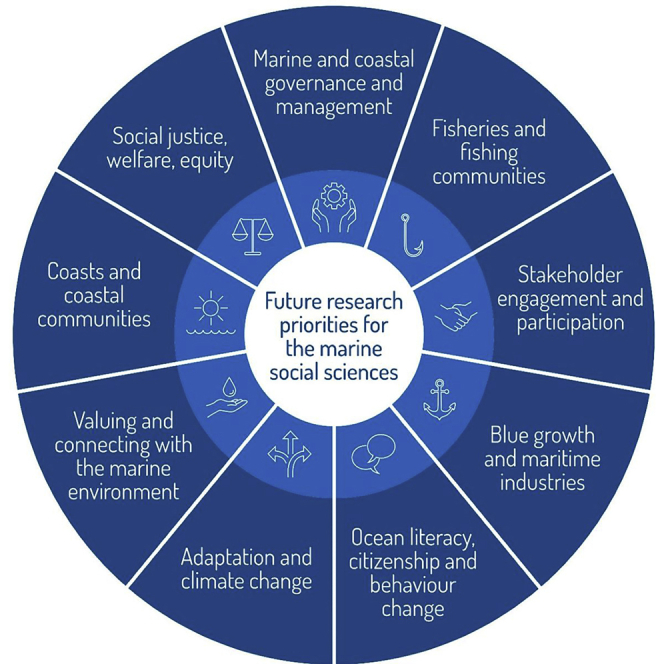

## Introduction

Marine environments are complex and dynamic socio-ecological systems. Today, climate change, resource exploitation, biodiversity loss, and myriad other pressures threaten marine systems from local to global scales (Halpern et al., 2015, 2019; Nash et al., 2017, 2020), with flow-on effects for human wellbeing (Nash et al., 2021). This has major implications for human uses and the future management of the ocean (Jouffray et al., 2020). Societal relationships with the ocean are complicated and multilayered influenced by a wide range of factors, including access to marine and coastal spaces, socio-demographic characteristics, dependency and connection with ocean spaces or resources, as well as the temporal and spatial variation of these drivers, as evidenced in a recent review of ocean perceptions research from Jefferson et al. (2021); this complexity therefore leads to s in diverse community perceptions and levels of ocean stewardship (McKinley and Fletcher, 2010; Fletcher and Potts, 2007). Coupled with the biophysical challenges facing the ocean, there is a growing acceptance that sustainable management and conservation rely strongly on public awareness and knowledge of ocean issues, active support, engagement and participation, and meaningful social mobilization to succeed (Jefferson et al., 2015; Bennett et al., 2017). While the natural sciences play a critical role in advancing our understanding of the physical aspects of the ocean and associated impacts, the social sciences provide the methodological approaches and theory necessary to advance our understanding of the human dimensions of the ocean (Claudet, 2021). The marine social sciences are thus paramount to addressing sustainability challenges now and into the 21^st^ century (McKinley et al., 2020).

Recent years have seen increasing calls to better document and understand human dimensions of the ocean and, crucially, incorporate this knowledge into policy and management (Claudet, 2021; McKinley et al., 2020; Bennett, 2019; Bennett et al., 2017). The substantial attention paid to social science recognizes the reality that *“no amount of natural science research will help us manage the oceans if we ignore the need for strategies that lead to evidence-based, participatory, and transparent management”* (Parsons et al., 2014, p1213). These calls are mirrored and further championed through a suite of international goals and obligations (e.g. the Sustainable Development Goals (SDGs) and the United Nations Decade of Ocean Science for Sustainable Development 2021–2030; Fleming et al., 2019; Ryabinin et al., 2019) and international policy drivers (e.g. Intergovernmental Panel on Climate Change, 2021). For example, the COP26 (Glasgow) in 2021, stated intention for an annual ocean-climate dialog and greater recognition of indigenous people and local communities is also suggestive of a greater focus on the human dimension in this forum.

The human dimensions of the ocean are best investigated and understood through the application of the marine social sciences (McKinley et al., 2020), a broad field of theoretical and applied research that seeks to examine, investigate, describe, predict, and understand human relationships with the global ocean, coasts, and seas (Moon and Blackman 2014). These include, but are not limited to, traditional social science disciplines such as psychology, sociology, and anthropology, but also encompass research in economics, governance processes, industry and blue growth, culture and heritage, health and wellbeing, and increasingly, arts and humanities (Bennett 2019; Bennett et al., 2017). Marine social sciences provide the tools and opportunities to identify and include the lived realities and perspectives of ocean and coastal communities in knowledge production and decision-making about ocean management and sustainability (Bavinck and Verrips, 2020).

An emerging community of work has led to an almost exponential growth in marine social sciences research in recent years (see for example, Jefferson et al., 2021). Research in the marine social sciences encompasses a wide range of research topics and themes, as well as a variety of approaches (see Bennett et al., 2017). Fisheries have long dominated the agenda of marine social sciences (Fleming et al., 2021; Steins et al., 2020; Bavinck et al., 2018). However, in recent years, other topics have garnered increasing attention (McKinley et al., 2019, 2020). Prominent topics include, but are not limited to, the integration of marine social sciences within natural science (Martin, 2020; Moon et al., 2021), community perceptions of marine-protected areas (Kelly et al., 2021a), and participation in marine spatial planning (Flannery et al., 2019; McKinley et al., 2019). Moreover, marine social sciences research has expanded to investigate the behaviors of diverse marine user groups Mackay et al., 2020; Stoll-Kleemann 2019), public and stakeholder perceptions toward marine environments (Potts et al., 2016; Jefferson et al., 2015), connections to the ocean (Kelly et al., 2021a; Borja et al., 2020; van Putten et al., 2018; McKinley and Fletcher, 2010), as well as the potential risks and benefits of exposure to marine and coastal environments (Borja et al., 2020; Department for Environment, Food and Rural Affairs, 2020; Fleming et al., 2019). Finally, recent years have seen an increased focus on connecting marine science and research in its broadest sense to policy and practice (see for example, Cvitanovic et al., 2018; Gilmour et al., 2015).

The expansion and diversification of marine social sciences has largely been enabled by a growing international community of practice, which includes the MarSocSci network (with chapters in the UK, Ireland, India, Italy, South Africa, and Australia), the International Marine Conservation Congress, the EU-based MARE consortium, the UK-based Greenwich Maritime Centre (which leads the biannual Society and the Sea Conference), and the IMBeR conferences (Future Oceans – IMBeR Open Science Conferences – IMBeR) and working groups (human dimensions IMECaN – Interdisciplinary Marine Early Career Network – IMBeR). The success of these emerging groups, networks, and events highlights the interest and benefits of knowledge sharing and collaboration within and beyond the marine social sciences. While not unique to the marine social sciences, we note that to date, most of this expansion has been focused within Western regions. Therefore, there is a need to establish cooperation and inclusivity between researchers and groups situated in, or conducting research on, regions in the global south (Maas et al., 2021; Tolochko and Vadrot 2021). While the continually developing interest in marine social sciences is both encouraging and necessary, the rate of expansion is perhaps outpacing the capacity to build a truly cohesive and collaborative community, resulting in a research landscape which is disparate and fragmented (see for example, Jefferson et al., 2021). Moreover, despite the evidence of growth, natural and physical sciences continue to dominate the global marine science and policy discourse (van Putten et al., 2021). There is a need, therefore, to understand the current marine social sciences research landscape (and its context in relation to the broader marine science landscape) and carry out something of a stock take so that priorities for the future can be identified (i.e. as explored by McKinley et al., 2020).

In light of this, this paper draws on the perspectives of global experts to generate prospective thinking for effective marine social sciences research and practice into the future, and aims to further awareness of the role and value of marine social sciences for global management and governance of marine environments. Building upon insights from the international marine science community, we highlight avenues for potential interdisciplinary collaboration (i.e. among and between marine social and natural science disciplines) that can inform human use and management of our oceans. In doing so, we expand upon research that has explored and discussed the development of marine social sciences (e.g. McKinley et al., 2020; Bennett 2019). Specifically, we advance the understanding of the challenges and opportunities facing the marine social sciences community and conduct a horizon scan to identify current trends and future directions, with the aim of developing a comprehensive global research agenda for marine social sciences. We seek to address four questions:

1. What are the current trends in marine social sciences research, and how do these vary globally?
2. How can the growth and development of a global marine social sciences research agenda be enabled?
3. What are the challenges and barriers facing marine social sciences?
4. What are the future priority areas for marine social sciences research?

### Methods

#### Questionnaire development

To elucidate the current trends, and critical gaps in relation to the marine social sciences, we adapted established horizon scanning methodologies. Horizon scanning is a participatory research approach that provides a structured and systematic methodology to elicit knowledge gaps and emerging issues by drawing upon the experience or knowledge of those involved in the process. For example, in relation to coastal and marine systems, horizon scanning approaches have already been utilized to develop research priorities relating to the management of coral-dominated marine-protected areas (Cvitanovic et al., 2013), recreational fisheries (Holder et al., 2020), and coastal management (Rudd and Lawton 2013).

Given the labor-intensive nature of horizon scanning approaches, and the geographic spread of marine social scientists, we modified existing approaches for simplicity, and to maximize response rates. Specifically, an online questionnaire was developed to obtain responses from across the global marine science research community. The questionnaire comprised of two sections, each containing a combination of open and closed answer format questions. Questions in section 1 were developed to understand the background of each respondent, particularly in relation to their previous experiences within the marine sciences and their current areas of research. Section 2 then posed a series of open-ended questions to elucidate the respondents’ views on the existing knowledge gaps and future areas of research for the marine social science community. Open-ended questions were used in section 2 of the survey to ensure that responses were not limited in any way (i.e. by discipline, geographic area, scale, etc.) and to ensure that responses were as broad as deemed necessary by the respondents. This approach is typical within horizon scanning methodologies and allows research gaps to emerge directly from the experience and knowledge of diverse respondents.

Ethical approval was obtained through Cardiff University’s Ethical Review Process in May 2020. The questionnaire was piloted through the authors’ professional networks prior to final dissemination. In total, five contacts from two countries provided feedback on the draft survey and minor textual changes were made based on the feedback from this process to improve clarity in the wording or the questions and remove ambiguity.

Respondents were recruited using a self-selection, convenience-based sample approach (Bryman, 2012), and criteria for inclusion were provided at the onset of the online survey so that respondents could clearly assess their eligibility to participate. However, as the survey was launched as an open call to all marine researchers globally, the potential for non-response bias was considered and addressed to mitigate any potential influence on recruitment and results as much as possible. First, to maximize geographical reach, the survey was shared and promoted online through diverse means. The survey was disseminated via direct emails to personal networks (i.e. via email to institutional mailing lists) and established marine science networks (e.g. including the Marine Social Science Network, the Royal Geographical Society's Coastal and Marine Research Group). The survey was also shared via social media (i.e. Twitter), as social media has been shown to be an effective recruitment strategy for online surveys (McRobert et al., 2018). Second, the survey was launched online initially for a duration of two weeks, but this was later extended to an additional 4 weeks (a total of 6 weeks, between May and July 2020) to enable increased uptake and participation. Third, the survey questionnaire itself communicated the aims of the survey, details about the researchers conducting the study, and information about participant confidentiality, which evidenced the legitimacy and value of the survey research to potential marine science and research participants.

In terms of expected audience, the survey targeted individuals working across the diverse disciplines of the marine social sciences (as explored by McKinley et al., 2020). This included, but was not limited to, marine researchers working within related disciplines of psychology, sociology, anthropology, and political science, among others. By sharing the survey through online networks, including targeted promotion of key groups known to the authors, we sought to facilitate the inclusion of respondents and perspectives from the global south to ensure that results were as broadly representative as possible.

#### Data analysis

##### Qualitative analysis

Open-ended questions were used to collect data across a number of topics. All qualitative data collected through the online questionnaire were analyzed manually adopting an emergent, inductive coding approach (Glaser and Strauss 1967; Charmaz 2008). First, the data were broadly coded against each of the four research questions (see section 1). Second, thematic coding of the data was undertaken to develop a coherent set of key themes. This involved the identification and interpretation of patterns or “themes” in the dataset. Emergent categories were developed and revised through a repeated review process to ensure its validity and relevance (Fleming and Vanclay, 2009; Marshall et al., 2011). Content analysis was then used to transform the qualitative data into categorical data. Content analysis quantified the content of the surveys in terms of the pre-determined themes or categories identified through inductive coding (Bryman, 2016). This enabled prioritization of key themes and further statistical exploration. The qualitative analysis provided additional insight into the global marine social sciences landscape, including current challenges and future priorities. The key themes identified are discussed in parallel to the quantitative analysis, with relevant quotes presented in italics to support the discussion as appropriate. The analysis was checked and agreed between two members of the author team to ensure satisfactory agreement of codes and themes.

##### Quantitative analysis

Responses to the survey questions were categorized to themes and collated, and demographic data, such as career stage, location, background, and type of scientist were collected for quantitative analysis. A Pearson’s Chi-square statistic was used to assess homogeneity and independence across groups within the sample. This enabled us to explore for any statistically different variations (testing for significance at an alpha value of 0.1 and 0.05; Brereton, 2020). Specifically, we explored if there was a significant difference across the demographic data and trends in marine social science research and the challenges and enablers for marine social science research (research questions 1 and 3). While inferences can be made from the survey data alone, the quantitative analysis can provide statistical backing to refute any null hypothesis that there is no relationship between any variables. However, it should be noted that this statistical test is highly sensitive to sample size and given the relatively small sample size and in some cases high dimensionality of categorical data (i.e. multiple possible responses for a question), there is a chance that some statistical differences may not be picked up with this test.

## Results

### The global marine social sciences community – Understanding the respondent profile

A total sample of 106 responses was collected through the online questionnaire—a summary of the respondent profile is presented in Table 1. The majority of respondents were early-career researchers (i.e. ECRs, including those in undergraduate levels to up to five years post PhD; 64%). Only 27% were in mid- or late career stage (18% and 9%, respectively). This perhaps reflects the relative youth of marine social sciences as a discipline and community of research, or simply may be an indication that ECRs are more active in their engagement in the platforms which were used to recruit respondents.

**Table 1:** Respondent profile (N = 106)

	N	%		N	%
**Continent**			**What sector do you work in?**		
Europe	44	42	Education/Academic Research	**72**	68
North America	29	27	Consultancy Research	7	7
Central and South America	7	7	Government and policy	9	8
Asia	6	6	NGO/Charity (e.g WWF)	7	7
Africa	5	5	Funding Body	1	1
Australia and New Zealand	14	13	Industry	1	1
**Employment status**			**Background**^a^		
Permanent/Full-time paid work	41	39	Biology	62	58
Permanent/Part-time paid work	1	1	Chemistry	4	4
Fixed term/Full-time paid work	20	19	Physical Geography	11	10
Fixed term/Part-time paid work	5	5	Human Geography	27	25
Retired	2	2	Law	8	8
In education	24	23	Politics	11	10
Unemployed (Seeking work)	7	7	Economics	7	7
Not in paid employment (not seeking work)	1	1	Arts	5	5
			Humanities	13	12
**Career stage**			Psychology	4	4
Undergraduate student	2	2	Sociology	19	18
Postgraduate student (Masters)	13	12	Anthropology	19	18
Postgraduate student (PhD)	31	29			
Early – up to 5 years post PhD	22	21	**Type of scientist**		
Mid – 6–20 years post PhD	19	18	A natural scientist	30	29
Late – over 20 years	10	9	A social scientist	38	36
			Both	37	35

a Note that respondents could select more than one option to this question and there were some missing cases for this question. Percentages have been calculated based on total sample size (N).

Most respondents indicated a background of education/academic research (68%). Over 70% of respondents indicated they had some physical or natural science background (e.g. 58% of respondents had a background in biology, Table 1). Finally, our respondents were geographically unevenly spread, with the majority of respondents based in Europe and North America (i.e. the Global North; 42% and 27%, respectively).

Analysis was also undertaken to understand the relationship between career stage, type of scientist, and geographical location. Career stage was significantly different across scientist types *X*^2^ (12, *N* = 105) = 29, p = 0.0039). Social scientists or those identifying as both a natural and social scientist were earlier in their careers, in comparison to scientists who identify as natural scientists who were more likely to be mid-career scientists. There was no significant difference found between the type of scientist and location i.e., there were not more marine social scientists in a particular geographic area. However, as illustrated in Table 1, the survey sample was dominated by respondents from North America, Australia and Europe, with considerably fewer respondents from Asia, Africa, Central, and South America (see SM1 and SM2 for more information).

### Current trends and global variations in marine social sciences research

Respondents were asked to provide details of their main area(s) of research (N = 103). As this survey focused on the social sciences, topics that fell outside of this realm (e.g. ecological research) were removed from the analysis (19 responses). Using the emergent coding process described above, analysis highlighted nine key research areas (Table 2). If responses were relevant to multiple areas (e.g. governance of the blue economy agenda), these were coded to multiple themes as appropriate (i.e. governance of the blue economy agenda would be coded to both blue economy and governance). The most popular topic area was governance and decision-making (23%), followed by participation and engagement (14%) and the socio-cultural value of marine and coastal environments (12%). The other remaining topics included: ocean literacy, area-based management, fishing industry and communities, blue economy, coastal communities, and “other”. Table 4 provides a further description of each topic with examples from the survey.

**Table 2:** Current research being undertaken within the field of marine social science (N = 87; 120 individual responses)

Categories	Description	% Frequency	Examples
1. Governance, management and decision-making	Includes research on marine and fisheries governance (e.g. understanding governance processes, evaluation of interventions, decision-making, and integration of knowledge into policy and practice). Encompasses all topics, except area-based management.	23%	• Marine Spatial Planning • UN Sustainable Development Goals • Fisheries management (e.g. community-based fisheries management).
2. Participation and engagement	Research into stakeholder and public engagement, and participation in research and policy with the aim of delivering effective marine and coastal management and decision-making.	14%	• Citizen science • Indigenous knowledge • Power relations
3. Socio-cultural value of marine and coastal environments	Examination of the value of marine and coastal environments—comprising monetary and non-monetary values. Includes frameworks such as ecosystem services and natural capital.	12%	• Natural and cultural heritage • Health and well-being • Economic valuation and modeling
4. Ocean literacy	Related to the levels of ocean literacy at various scales. This includes research into awareness, knowledge, attitudes, communication, behavior, and activism.	10%	• Coastal stewardship • Public awareness and perceptions of the marine and coastal environment • Public perceptions of marine industry
5. Area-based management	Research into area-based management tools—including evaluations of the costs and benefits of interventions.	10%	• Community-based management • Community livelihoods in MPAs • Design and evaluation of MPAs
6. Fishing industry and communities	Includes all topics related to the fishing industry (commercial, artisanal, small scale, and recreational) and communities. This includes the value of fishing and impacts on the supply chain, in addition to the welfare and wellbeing of fishing communities.	9%	• Recreational fisheries • Gender and fisheries • Food and nutrition security
7. Blue economy	Related to the Blue Economy, Blue Growth, and topics associated with all types of maritime industries (e.g. tourism, recreation, and renewable energy).	9%	• Regional economic development • Ocean clusters • Community chains
8. Other	Miscellaneous topics described by respondents.	11%	• Public access to marine and coastal spaces • Invasive and/or non-native species • Modeling of socio-ecological systems

### Enabling the growth and development of a global marine social sciences research agenda

Respondents were asked to list up to three enablers which may assist in increasing the uptake of marine social sciences research in ocean decision-making and help to overcome barriers. Over half of respondents answered this question (53%), resulting in 156 listed enablers (see Table 3). The most commonly discussed enablers were: the promotion of marine social sciences (35/156; 28%) and engaging stakeholders and enlisting champions (24%). Respondents highlighted the benefits of using “*Informed mediators between scientists and policy makers”* (ID20) and “*Recruiting social scientists into decision-making roles in government”* (ID79). This was in addition to the importance of champions advocating for marine social sciences across the science-policy landscape. For example, “*Individual leaders that keep banging on the door to make change”* (ID76) and *“Influential ‘cheerleaders’ - politicians pushing for recognition of marine social science”* (ID81).

**Table 3:** Enablers for increasing the uptake of marine social science research in ocean decision-making (N = 57; 156 individual responses)

Theme	% Frequency
Promote marine social sciences	28%
Engage stakeholders and enlist champions	24%
Build interdisciplinary teams	21%
Working at the science-policy interface	15%
Provide training and capacity building	8%
Improve funding	7%
Broaden the marine science toolkit (including within marine social sciences)	3%
Ocean literacy	3%
Communicate and promote marine social sciences	28%
Other e.g. understanding vulnerability	10%

Participants also highlighted the importance of interdisciplinary teams in the marine social sciences (21%), emphasized by a number of respondents—“*Working together in multi-disciplinary teams” (*ID21). Furthermore, the need to increase understanding of marine social sciences to support interdisciplinarity was discussed—this was exemplified by one participant who emphasized a need for “*Encouraging social scientists to communicate in an understandable way with those who traditionally make or influence decisions (including natural scientists)”* (ID79). The need for improved communication was also reflected by participants, who identified a need for *“Social Media presence”* (ID33) and an *“Awareness campaign”* (ID36), with the aim of further raising the profile of various dimensions of marine social sciences. Finally, while mentioned less frequently, participants also outlined a range of enablers which may assist in increasing the uptake of marine social sciences research in ocean decision-making and thus help to overcome identified barriers (summarized in Table 3).

**Figure 1:**
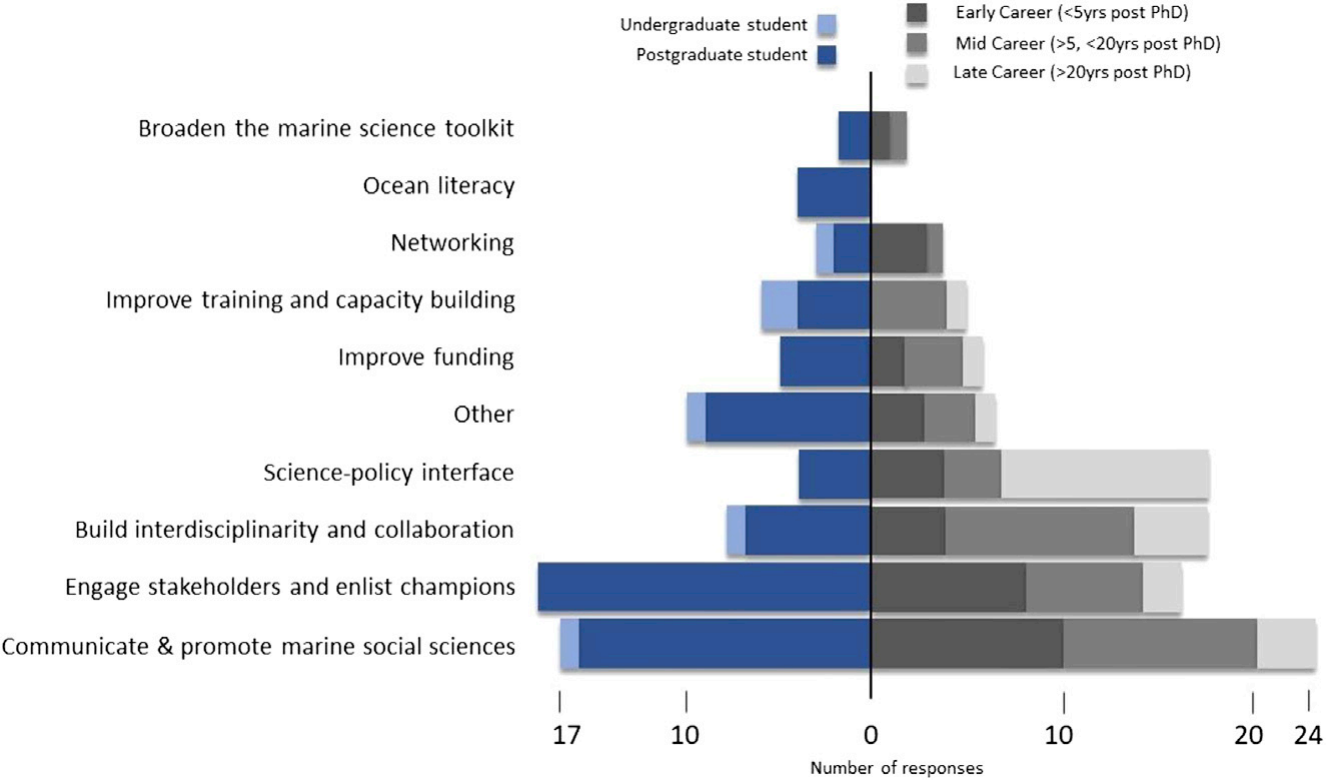
Career enablers by career stage (total number of responses = 178) The undergraduate and postgraduate students are shown in shades of blue and post PhD in shades of gray.

Additional analyses were undertaken to examine whether the identified enablers varied with different respondent characteristics. There were significant differences in enablers across the various career stages (*X*^2^ (45, *N* = 178) = 79, p = 0.0014; Figure 1). For example, working at the science-policy interface was predominantly listed by those later in their career (e.g., those 20 years post PhD). This contrasted with enablers such as engaging stakeholders and enlisting champions, and promotion of marine social sciences, which were highlighted by participants who were earlier in their careers (e.g. postgraduate students). Surprisingly, providing training and capacity building was not listed as an enabler by ECRs but was listed by all other career stages. Another stand out difference is that only postgraduate students identified “ocean literacy” as an enabler for increasing the uptake of marine social science research in ocean decision-making.

### Challenges and barriers facing marine social sciences

Respondents were asked to list up to five challenges (or barriers) that could prevent or limit the uptake of marine social sciences research in ocean decision-making. Participants identified seven types of barrier facing the marine social sciences (N = 66, 252 individual responses; see Table 4). They included: (i) opportunities and capacity, (ii) the relationship between marine social sciences and other disciplines, (iii) visibility and recognition of marine social sciences, (iv) marine social sciences theory and application, (v) engagement with policy and decision-makers, (vi) engagement with stakeholders and the public, and (vii) other.

**Table 4:** Summary of the challenges or barriers to marine social sciences (N = 66; 284 individual responses)

Name of barrier	% Frequency
Opportunities and capacity	21%
The relationship between the marine social sciences and other disciplines	20%
Visibility and recognition of marine social sciences	18%
Marine social sciences theory and application	12%
Engagement with policy and decision-makers	11%
Engagement with stakeholders and the public	10%
Other	8%

Of the seven challenges, three were mentioned most frequently in the survey responses. The first concerned the availability of opportunities and capacity within the field. A quarter of survey responses highlighted this as a key barrier (21%). Participants discussed the lack of funding, jobs (i.e. availability of relevant roles and permanent positions), leadership opportunities (e.g. leading projects and initiatives), training opportunities (e.g. lack of integration of marine social sciences into formal education, and paucity of external training courses), and limited research capacity (e.g. lack of expertise within organizations, and poor research coherence). Funding was mentioned most frequently, and respondents perceived that less funding was available for interdisciplinary and marine social sciences research, particularly in comparison to natural science research counterparts. As exemplified by one participant, marine social science is a “*New ‘discipline’ so there is lower* *funding* *except for* *support* *of traditional marine science”* (ID26).

The second barrier related to the relationship between the marine social sciences and other disciplines (20%). Respondents indicated that there were often misconceptions about the broad field of marine social sciences and it affected the success of marine social sciences and the effectiveness of collaboration with other disciplines. For example, some respondents believed that there is a perception *“… that the job of social scientists is to help natural scientists. communicate and involve stakeholders”* (ID17) and “… *that our work is ‘common sense’”* (ID68). Responses also focused on the *“Dominance of natural sciences with reductionist approaches”* (ID7) and voiced that “*Funding* *opportunities [are] still much more dominant for natural marine science”* (ID21). They also highlighted the existence of siloes and a lack of integration and collaboration between and within disciplines as a barrier, with one participant stating that they were *“Very much working on silos or when in partnership boxed to do the ‘social’ work”* (ID66).

Third, respondents emphasized the lack of visibility and recognition of marine social sciences as a key barrier (18%). They perceived that marine social sciences still needs to gain legitimacy and voiced that they often felt the need to have to prove the value of their discipline(s) to other groups (e.g., to researchers in other disciplines, to policymakers, stakeholders, as well as to the public). One respondent stated that they felt that marine social sciences were *“Undervalued/underrated within science fields (soft science) and for policy makers”* (ID22). Furthermore, that there was a need for *“Gaining legitimacy among non-academic institutions and communities (e.g., government, industry, communities)”* (ID49). They also stressed the importance of raising awareness and understanding of marine social sciences. Multiple respondents commented on the broader lack of awareness of the importance of the field of marine social sciences and its role in the wider system of research and practice; e.g.: *“Lack of understanding that ocean ecosystems include human social and cultural systems”* (ID7); “*Misunderstanding of the breadth of the social sciences…[and] the vastly different theoretical underpinnings of these fields*” (ID37). Respondents also saw communication and language as a barrier to gaining visibility and recognition and discussed the need for targeted and increased communication of marine social sciences, as well as more accessible language and terminology. One respondent highlighted that there was “*Poor public communication of social science issues—jargon-filled, long-winded, abstract communications”* and highlighted the need for *“Communicating research to those who could use it”* (ID7). Finally, while mentioned less frequently, participants also outlined a range of other challenges or barriers to marine social sciences (summarized in Table 4). As before, additional analyses were undertaken to examine whether the identified enablers varied with different respondent characteristics, with no significant difference identified between barriers and respondent socio-demographic characteristics.

### Future priority areas for marine social sciences research

Respondents were asked to identify up to five research priorities for marine social sciences globally. A total of 301 individual responses were gathered from this question. Initial thematic analysis found that not all responses aligned with research themes, instead they highlighted a range of perspectives on the operationalization of marine social sciences, including collaboration, outreach, and governance. Therefore, the findings were separated and categorized as follows: (i) operationalization of marine social sciences and (ii) research priorities for marine social sciences research.

#### Operationalization of the marine social sciences

Respondents highlighted five main priorities related to the operationalization of the marine social sciences (see Table 5 for definitions of each). These were: (i) community involvement and ocean literacy (36%), (ii) governance and decision-making processes (30%), (iii) research landscape (18%), (iv) marine sectors and research areas (17%), and (v) communication and capacity building (10%).

**Table 5:** Description of emergent themes relating to priorities

Theme	Definition and example of topics included within the theme
Community involvement and ocean literacy	Includes all topics relating to ocean literacy, marine citizenship, and behavior change; community involvement and co-design; coastal community engagement and adaptation; inclusion and understanding of diverse values and knowledge within research and policy making.
Governance and decision-making processes	Includes all topics relating to research led governance and decision-making (including understanding governance processes, how people make decisions and how knowledge can be integrated into policy and practice) across the science-policy-practice interface.
Research landscape	Includes all topics relating to research planning, development, and implementation including: Funding, Interdisciplinarity and collaboration; evaluation of research impact.
Marine sectors and Research Areas	This includes topics relating to specific marine sectors and research topics/areas mentioned by respondents, including: fisheries, blue growth, marine-protected areas, link between ocean and human health, social justice and equity.
Communication and Capacity Building	Includes all topics relating to science communication, communication with communities but also across sectors and research communities; skills and capacity building.

**Figure 2:**
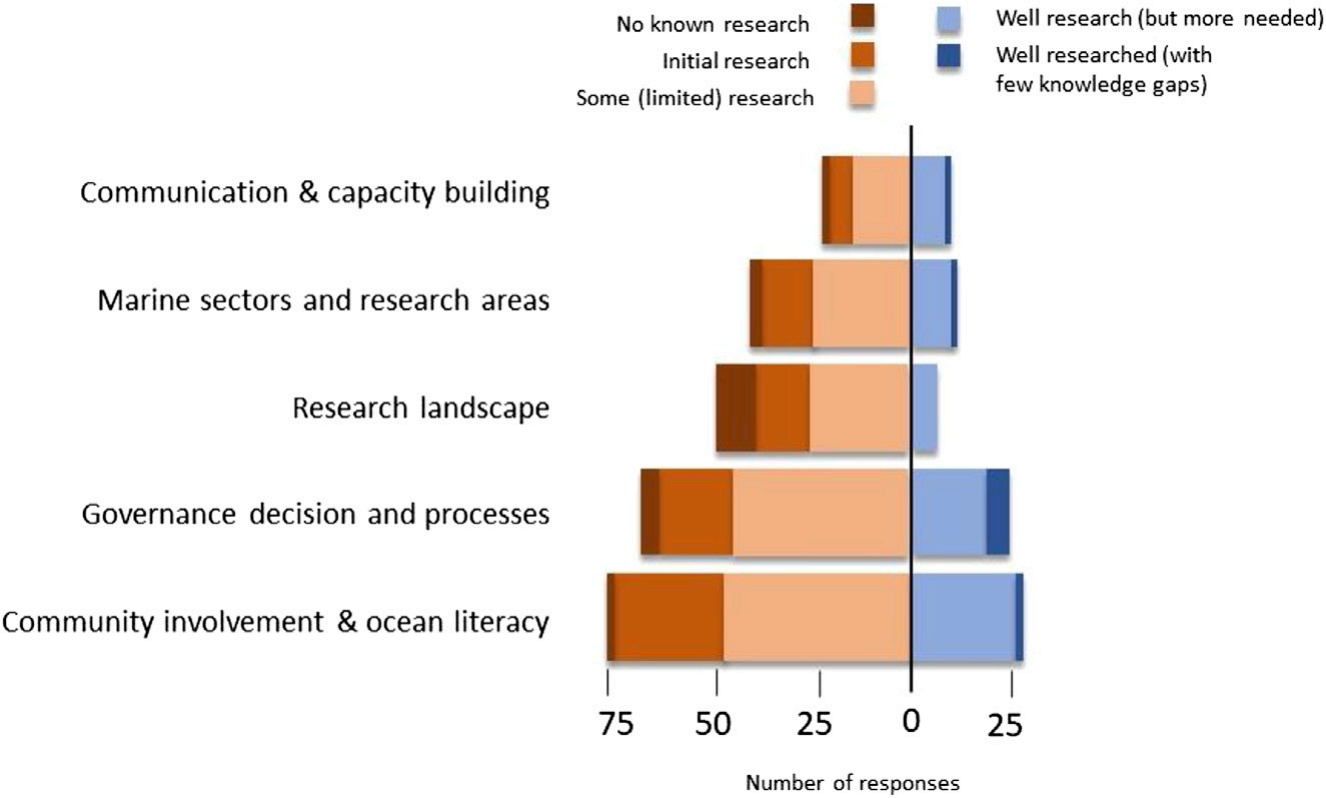
Research theme by perceived level of information available (total number of responses is 324) The areas that are well researched are shown in shades of blue and those less well or not research are shown in shades of red.

As part of the question, respondents were asked to indicate their perceptions on the current research status of each of the priority areas (i.e. *no known research in this area/topic, initial research in this area/topic, some research in this area/topic, well researched but more research is needed*), with a view to identifying priority areas for the marine social sciences (see Figure 2).

From the quantitative analysis, there was a significant difference between the research priority themes across the level of current research being conducted for each theme (*X*^2^ (16, *N* = 351) = 25.2, p = 0.066). For instance, the lowest level of research was attributed to the theme “research landscape”; this theme proportionally had the lowest score for “well researched with few knowledge gaps”. This indicates that gaps and challenges remain, with opportunities for improvement and the development of a coherent and supportive research landscape (e.g. appropriate funding, provision of training, and capacity building) to effectively further marine social science research. In addition, it should be noted that none of the overarching themes were identified as being “well researched with few knowledge gaps” suggesting that all areas of marine social sciences research would benefit from further effort and data collection.

For these thematic priorities, we explored differences in themes across specific socio-demographic characteristics (type of scientist, career stage, employment status, and geographic region—at the continental scale). There were no significant differences found across any of the groups (see Tables SM3-6 for a detailed summary), with the highest proportion of research priorities related to community involvement and ocean literacy across all groups. While the differences are not significant, detailed examination of the data indicated some observations that were of note. No significant differences were identified between employment status; however, those in fixed term employment more frequently mentioned topics relating to “research landscape” compared to any other group. Finally, no significant differences were identified across continents. However, the analysis did indicate that participants in Africa had proportionately more research priorities relating to community involvement and ocean literacy than other areas, whereas Central and South America more commonly related topics associated with governance and decision-making processes.

#### Future research directions and priorities for the marine social sciences

**Figure 3:**
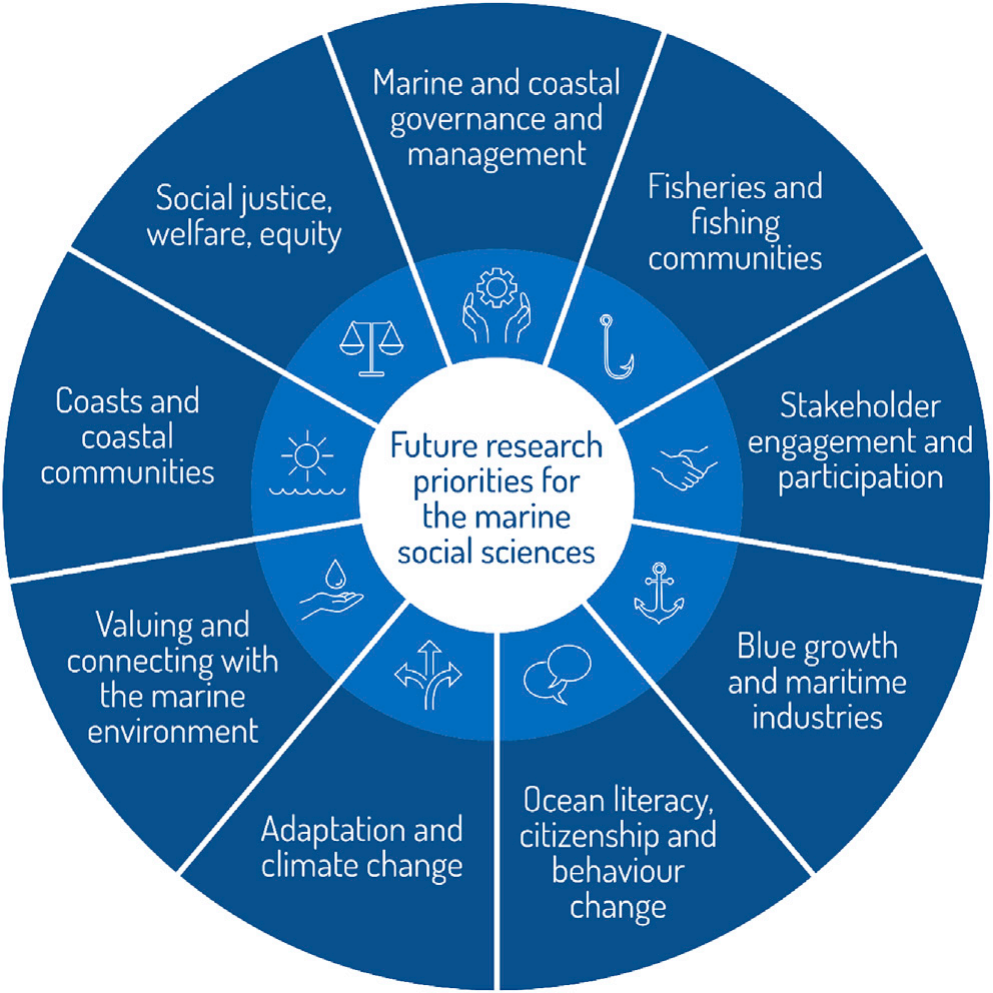
Future research priorities for the marine social sciences

In-depth analysis was conducted to explore the research-specific priorities listed by respondents. 62 respondents provided research-specific priorities (resulting in a total of 186 individual responses); which are summarized in Figure 3 and in more detail in Table 6.

**Table 6:** Future research priorities for marine social sciences (N = 62; 186 individual responses)

Research Theme	Description	Examples
Marine and coastal governance and management	Includes all topics relating to marine and coastal governance and management, including impacts of planning and spatial management interventions and development and delivery of policy across all scales. Also includes themes relating to the development of governance, legislation, and decision-making for all aspects of marine and coast, including management of maritime industries.	• Understand the barriers that constrain effective governance of oceans • Examine how non-ecological values be incorporated into environmental impact assessments • Development of effective and monitoring evaluation for ocean governance (e.g., Marine Protected Areas) • Investigate how to include new perspectives and disciplines into management
Fisheries and fishing communities	Includes topics relating to the value (both monetary and non-monetary) of all scales of fisheries (including commercial, artisanal, small scale, and recreational fisheries), governance and management of fisheries, the welfare and wellbeing of fishing communities, as well as direct and indirect value and impacts across the supply chain.	• Examination into inclusion of fisher knowledge in science and management • Improve understanding fisher behavior/choice strategies • Investigate role and challenges for fisheries and aquaculture in future global seafood food security • Research into human rights in fisheries industry • Improve/expand data collection on small-scale fisheries and associated industries, including post-harvest (e.g. connectedness)
Stakeholder engagement and participation	Relates to topics including methods of stakeholder and community engagement and development of best practice, and the need for meaningful stakeholder engagement across all sectors and aspects of community to deliver effective marine and coastal management and decision-making.	• Develop best practices for knowledge integration across different forms of knowledge generation (e.g. Traditional Ecological Knowledge, Local Ecological Knowledge and Citizen Science • Evaluate theory and link with practice associated with broad, multisector stakeholder engagement (e.g. network analysis to identify key stakeholders • Improve participatory monitoring systems in order to maintain optimized resource utilization by securing their sustainability • Understand ocean users, their knowledge and motivations, to effectively engage these communities in ocean planning and management
Blue growth and maritime industries	Includes topics relating to all types of maritime industries, including but not limited to tourism, recreation, marine renewable energy, aggregates, and also including themes relating to growth of these sectors, impacts on maritime workers, and extended communities and the governance and management of these systems.	• Investigate the feasibility of marine renewable energy and its potential implications for fisheries and coastal communities • Research into regional Blue Growth and how it can be defined •Study of power dynamics of Blue Growth •Examine the impacts of the circular economy on blue economy and sectors
Ocean Literacy, citizenship and behavior change	This overarching theme includes topics relating to the parallel concepts of ocean literacy and marine citizenship, and therefore includes environmental education, awareness-raising, and behavior change (e.g. understanding how to engender behavior change across different audiences and types of communities).	• Understand the drivers and effective strategies to elicit environmentally sustainable behavior • Understand how collective action is changing with global environmental change • Evaluation of public understanding of marine issues and how links to behavior • Research into conservation marketing
Adaptation and climate change	Relates to climate change and topics of adaptation, including but not limited to sea level rise, coastal erosion, ocean acidification, coral bleaching, as well as management and governance issues, such as managed realignment, shoreline management plans, and coastal defense. Blue carbon initiatives are also included within the overarching theme of blue growth.	• Understand most effective approaches to adaptation and mitigation • Study of impacts of climate change on socio-ecological marine systems • Research into plans for changes in climate, resources, and ocean-safety
Valuing and connecting with the marine environment	Includes research relating to societal and individual connection with the marine and coastal environment, and understanding, measuring, and monitoring the values (economic, social, and cultural) attributed to the seas and coastline.	• Investigate and understand the socio-cultural values that individuals and communities attach to the marine environment • Quantification and scenario analysis of Ecosystem Services (e.g. how to optimize resource exploitation and Ecosystem Services). • Investigate the role that values and culture play in marine resource management • Develop a different language of how we speak about the communicate about the value of the marine environment (i.e. legitimize intrinsic values)
Coasts and coastal communities	Research the importance of recognizing the value of coastal spaces, alongside that of the marine (i.e. under sea/ offshore) environments.	• Understand the intersection between coastal values and environmental significance • Develop an understanding of the importance of blue spaces for human health and wellbeing • Study of land to sea interactions and connected policy development • Conduct a global review and evaluate the barriers and solutions to effective regenerative development for coastal cities
Social justice, welfare, equity	Includes but is not limited to topics relating to access, equity, and equitability relating to marine and coastal resources, as well as gender equality (e.g. women in maritime industries or the role of women in fishing communities), welfare of maritime workers (e.g. migrant crews or those working offshore).	• Improve understanding of equitable management (e.g., for ocean resource use and access) • Investigate the equity issues around access to and use of marine resources for health and wellbeing • Understand the role of women in fisheries • Examination of livelihood strategies of coastal communities and how these can be strengthened.
Other	Includes areas not covered in any of the above categories.	• Examination of research impact in marine social science • Research into transdisciplinary knowledge creation in the Marine/Ocean Space • Develop robust socio-ecological indicators for countries globally • Develop and improve effectiveness of science to policy to implementation pathways

## Discussion and recommendations

The marine social sciences are increasingly recognized as key to addressing the challenges facing our global ocean, seas, and coasts (Fleming et al., 2019; Ryabinin et al., 2019). This paper presents a global assessment of the current status and trends in marine social sciences research, highlighting a clear need to build upon existing research effort, as well as the design and use of new interdisciplinary knowledge that can support ocean sustainability goals. With this in mind, the paper has identified a series of future priorities, both research-focused and operational, to provide much-needed direction for the continued development and expansion of marine social sciences globally. However, numerous challenges and barriers remain, which may affect efforts to solidify the role of the marine social sciences within the broader field of marine science and integrate the marine social sciences across the research-policy-practice interface.

### Extent and significance of the marine social sciences

The field of marine social sciences is often perceived as a relatively nascent community (Bennett, 2019; Parsons et al., 2014). However, this study reaffirms that recent development and expansion of marine social sciences is underpinned by an active, growing, diverse, and interdisciplinary network of researchers and practitioners (McKinley et al., 2020; McKinley, 2020; Bavinck and Verrips, 2020). While there are signs of improved recognition of the integral role of marine social sciences within decision-making and development of sustainable ocean governance across a multiplicity of scales and contexts (e.g. through the UN Ocean Decade), there is more to be done (van Putten et al., 2021).

Encouragingly, this study finds that the themes and priorities for marine social sciences identified by respondents are largely in line with the vision and aspirations set out by a broad suite of global ocean policy drivers. Clearly, while opportunity for improvement in these areas remains, this first attempt at a global assessment, while dominated by certain geographies, infers a substantial and ever-growing field of marine social sciences evidence and data. Furthermore, while recognizing the geographical bias of the data collected, this study found reasonably high levels of agreement as to where existing research is adequate and where additional effort is required. It is possible that this result may indicate synergies, and reflect the prevalence of collaboration and interaction between early and late career stage researchers within the marine social sciences. Consequently, there may be opportunity to leverage these collaborations to guide the direction of marine social sciences research into the future. Although there are, of course, gaps in knowledge, methodological approaches, and geographical extent remaining (for more, see Jefferson et al., 2021), it is abundantly clear that the marine social sciences community is making much-needed contributions to design and delivery of sustainable and effective ocean management and decision-making. There is, therefore, a strong foundation on which future marine social sciences research activities can be built.

### A pro-active research framework for the future of marine social sciences

This study has developed a research framework for the future of marine social sciences. This framework provides a foundation for the development of a marine social sciences research agenda, building on earlier work carried out by McKinley et al. (2020). Harvesting research questions and topics from a diversity of horizon scanning exercises and articles is an important first step toward helping the scientific community to generate an overview the emerging range of challenges across regions, ecosystems, and scales. While we recognize that this study is not exhaustive and is certainly not representative of all geographies or career stages, the research priorities for the marine social sciences identified here serve as a much-needed starting point to stimulate global discussion and action during this important UN Ocean Decade, and will inspire discussion across sectors, and further investigation within public and private research institutions, academic institutions, NGOs, and industry (Wisz et al., 2020).

Moreover, the list of research priorities presented in this paper (Table 6) highlights a number of specific challenges relating to ongoing ocean sustainability efforts, perhaps emphasizing the urgent need for this work. There is opportunity, therefore, for this paper to be used as a guide within global secondary and higher education, as well as those who establish and design research programs, to contribute to the much-needed shift from the historical dependence on natural and physical sciences, as identified and called for by many scholars (Jefferson et al., 2021; Gardner, 2021; Parsons et al., 2014). Additionally, the calls for interdisciplinarity observed in this study echo recent calls from others to foster connections and collaboration across sectors and actors, bringing in diverse perspectives from researchers, stakeholders, communities (including Indigenous Peoples), industry, natural resource management, and policy to work together in an integrated, inclusive approach toward achieving more equitable and sustainable ocean futures (McKinley et al., 2020; Bavinck and Verrips, 2020; Parsons et al., 2014).

Furthermore, the relatively large early-career researcher representation in the results of this study likely presents further potential and opportunity for innovation and collaboration within the marine social sciences. Achieving such potential requires supporting early-career graduates and researchers (e.g., improving access to training, funding, as well as institutional and practical support) and providing them with opportunities to network, collaborate, and innovate. Early-career researchers are well placed to innovate and champion new approaches to addressing critical research priorities, including those identified through this study; e.g. by providing novel perspectives and highlighting different needs and interests. Collaborations with and between early-career stage researchers are likely to improve research quality and diversity within the marine social sciences (Kelly et al., 2021a; Keynejad et al., 2021; Pannell et al., 2019), particularly in the context of agenda-setting and the development of longer term initiatives (e.g. Brasier et al., 2020).

Finally, while this paper initially set out to present an overview of future research priorities, many factors influencing the operationalization of the marine social sciences community were highlighted at numerous points in the analytical process. The multiple calls for improved integration between natural and social sciences, and more recently, the aspiration of the UN Ocean Decade to deliver a transformational relationship between society and the ocean, are perhaps a signal of a “turning of the tide” with regards to the role of social sciences within the world of marine science and research. Crucially, however, this study highlights the opportunity to go further, and to recognize the value of true interdisciplinarity, building on inherent interconnectivity and overlaps between some areas of the marine social sciences, and arts and humanities-related disciplines (seen, for example, through the UK’s Valuing Nature Programme). Realizing this, demands systemic change (i.e. in terms of funding calls, design, and development of pathways to impact within decision-making) and a shift in how inter-, and indeed, transdisciplinary research is valued and championed (Cvitanovic et al., 2021a; Blythe and Cvitanovic, 2020; Kelly et al., 2019). These themes, and more, are discussed further in the next section.

### Overcoming challenges and barriers

The barriers and challenges identified in this study echo those reported by others, and continue to be problematic for the wider marine social sciences community (see e.g., McKinley et al., 2020; Bennett, 2019). These collective reflections emphasize central issues and the need for a coordinated approach to addressing shared problems. As such, this paper acts as a reminder of widely recognized barriers and challenges, and sets out a call to the community to consider how they can be addressed and navigated by working together. While navigating these barriers is in itself challenging, insights gathered from this study suggest that concerted effort in the following five areas would act as an initial platform for change.

Firstly, an overarching challenge identified through this study is the acknowledgment that the marine social sciences have experienced a poor reputation, when compared to natural and physical science counterparts. This reflects the work of numerous authors, who have emphasized the importance of addressing these reputational challenges (McKinley et al., 2020; Bennett, 2019). These include: the disregard for social science methodologies; limited understanding of the rigor, robustness, and validity of social science research and; indeed, its essential role in delivering effective ocean governance and management, which is currently prohibited by a lack of effective pathways to policy impact or processes for meaningful knowledge exchange between diverse actors (Cvitanovic et al., 2021b; McKinley et al., 2020; McKinley, 2020; Martin, 2020; Bennett et al., 2017). In this global study, we have identified several potential solutions to this reputational misunderstanding experienced across the marine social sciences community.

Secondly, our study highlights the need to prioritize capacity building and training. This will be essential for promoting best practice and delivering high-impact science that will inform conservation, policy, and sustainable development. It may also help to reduce the occurrence and risks of “parachute science” and similar challenges (Asase et al., 2022). Additionally, targeted capacity building will be important in responding to the growing number of non-social science experts now interested in, and indeed conducting, social science-inspired research. This will ensure an upskilling and training of non-social scientists interested in becoming more interdisciplinary in their own research and practice (Jefferson et al., 2021; Gardner, 2021; McKinley et al., 2020; Martin, 2020; Moon et al., 2019). Alongside this, the insights gathered through this study recognize the call for marine social sciences “champions”, who can advocate for the inclusion of social science research for the ocean.

Drawing on the points above, the third observation from this study is that there must be concerted effort to achieve equity, inclusivity, and diversity across marine social sciences. While this study is perhaps limited in its geographic scope, there was clear recognition from respondents that marine social sciences must move beyond existing geographical boundaries. Historically, marine social sciences have perhaps been grounded in research that was predominantly reflective of western ideologies and knowledge types. However, recent years have witnessed a change in discourse with more and more emphasis on the importance and need for transdisciplinarity and the inclusion of diverse voices, actors, and traditional knowledge systems for sustainable ocean governance (Fischer et al., 2021; Edwards et al., 2021; Bennett et al., 2021; Smith et al., 2017). In addition to considering who participates in marine social sciences research, there is also a need to be aware of who has the opportunity to develop marine social sciences research and application skills and become a “marine social scientist”. Building a community with inclusivity, equity, and diversity at its core therefore also requires access to funding for research and capacity building to those outside of the “usual suspects”, that transdisciplinary approaches are valued and championed, and that targeted training is made available to those in areas where there has been limited focus in the past (e.g. regions in the Global South). 10.13039/100014337Furthermore, the marine social sciences community can further lead the charge in terms of inclusivity by actively supporting early-career researchers, who are increasingly beginning their careers with interdisciplinary training and problem-solving skills, and thus have a key role in shaping the future of the marine social sciences (Kelly et al., 2021b).

Fourth, there is a clear need for integrated funding for inter- and transdisciplinary research that brings together researchers and practitioners from across the broadest definition of ocean science and research (e.g., see van Putten et al., 2021; McKinley et al., 2020). In exploring the disciplinary backgrounds of our survey respondents, this study indicates a trend where a growing number of researchers are moving across disciplinary boundaries (i.e. from natural sciences to social science-focused research) and engaging in interdisciplinary research (Table 3). This in itself may be a response to growing calls for increased interdisciplinarity within marine science and research (as seen in the goals of the UN Ocean Decade and called for in earlier studies, including McKinley et al., 2020). Interdisciplinary research brings together previously disparate ideas and concepts to produce novel ideas with high impact (Fortunato et al., 2018). While interdisciplinary research has been shown to garner less favorable funding results (Bromham et al., 2016), we recommend that funders draw on insights from this study which clearly illustrate that the marine social sciences community is not solely made up of “pure” social scientists—rather, there are many people within the community who have moved between research disciplines.

Interdisciplinarity is a strength and should be seen as such. It champions the inclusion of diverse world views and approaches. Furthermore, it can be a pathway to ensure meaningful engagement and contributions from all relevant, particularly those who have been historically alienated from or disengaged with ocean science and policy, thus ensuring social equity across all aspects of ocean science and decision-making (Bennett et al., 2021; van Putten et al., 2021). Fundamentally, it must be recognized that the challenges facing the ocean will not be solved by stakeholders one research discipline. Although there are inherent strengths of inter- and indeed, transdisciplinarity, there are also challenges within the types of capacity building and potential “blurring” of disciplinary boundaries suggested here (see Moon et al., 2019; Moon et al., 2021; van Putten et al., 2021). The provision of adequate funding and resources that can support the time and effort needed to foster networking and the development of collaborative relationships outside of traditional disciplinary siloes must therefore be a central component of any future funding frameworks (Cvitanovic et al., 2021a). Crucially, the marine social sciences must no longer be seen to be on the periphery of ocean issues or viewed as an add-on to projects—critical human dimensions insights must be incorporated from the outset of project planning and design to achieve sustainability outcomes. Funding calls must reflect this need.

Finally, although not marine social sciences in nature, it is important to note that broad environmental challenges will influence the direction of marine social sciences research into the future. It is therefore crucial that horizon scanning style studies are conducted regularly to sense-check the status of ocean sciences and the evidence and data needs of decision-makers—as well as to stimulate futures thinking (e.g. Kelly et al. in prep), in order to be able to predict, respond to, and prioritize the most pressing of challenges. While there are of course myriad “unknown-unknowns”, there is clearly a role for the global marine social sciences community in facilitating and shaping these kinds of transformational approaches to global ocean governance and sustainability. While this paper presents the results of the inaugural study to gather insight from the global marine social science community, there are limitations of the study that must be acknowledged as we consider its value in informing the direction of future research. Firstly, the authors recognize the inherent complexities in designing and disseminating global surveys of this extent; it is difficult to truly know the extent of the population, meaning choosing an adequate (and therefore, representative) sample size is a challenge. While the methodological approach adopted sought to minimize this, there is a clear geographical bias in our study which must be recognized and, where possible, addressed in future studies to ensure the marine social science discourse is representative of global research needs and priorities. It should be noted that, due to the lack of funding for this work, the survey was only available to participants in English, and was disseminated using a limited range of social media platforms and networks. While this paper nevertheless presents an initial insight into the global marine social science community, these limitations must be recognized and the dominance of the global north must be considered when interpreting the results. Future assessments would benefit from the availability of multi-lingual data collection tools, and also through distribution of these tools using a wider range of platforms to access those most commonly used in the global south (e.g. Facebook and LinkedIn, rather than Twitter).

## Concluding remarks and recommendations

This paper supports the findings of previous studies (e.g. McKinley et al., 2020; Bennett, 2019) and identified an active and apparently continually expanding marine social sciences community of researchers and practitioners. Furthermore, this paper describes a community which has, and continues to face, multiple challenges and barriers. However, by addressing the operational challenges identified herein and developing a comprehensive understanding of the existing research gaps and future priorities, the true potential of marine social sciences can be realized. Crucially, this paper presents a current overview of global marine social sciences research topics and provides much-needed insight into existing knowledge gaps and priorities. Drawing on insights from the international community of marine social sciences researchers, we can visualize a pathway toward a sustainable and fruitful marine social sciences community, through the following recommendations:

- **Establish a co-designed, transdisciplinary marine social science research agenda** that can respond to global priorities and is mobilized to address “wicked” problems currently facing the global ocean, coasts, and seas. This agenda must recognize the rich diversity of disciplines encompassed by the marine social sciences community and should draw on different skills, values, experiences, and knowledge types to facilitate meaningful and impactful expansion of the marine social sciences.
- **Increase the availability of integrated funding opportunities** which promote and support ocean science in its broadest sense—to ensure inclusion of marine social sciences research from across a range of disciplines from the outset.
- **Identify and enlist marine social sciences “champions”** from across the science-policy and science-innovation interfaces to advocate for marine social sciences research to be seen as an essential aspect of ocean sciences of the future. Crucially, these champions should represent different actors and knowledge types, and be demonstrative of the central tenets of **inclusivity, social justice, and equality** discussed above (see also Bennett et al., 2021).
- **Provide targeted and regionally specific training and capacity building** to i) enhance the reputation of marine social sciences, but also ii) increase capacity of marine social sciences across the science-policy-practice sphere, including within institutional and governance processes.

Undertaking these actions will be critical for unlocking and enabling the potential of the marine social sciences to contribute to sustainable, desirable, and equitable ocean futures for all.

## STAR★Methods

### Key resources table

**Table:** 

REAGENT or RESOURCE	SOURCE	IDENTIFIER
**Deposited data**		

Raw and Analysed Data	This Paper	N/A

**Other**		

Online Questionnaire	This Paper	N/A

### Resource availability

#### Lead contact

Further information and requests for resources and reagents should be directed to and will be fulfilled by the lead contact, Emma McKinley (mckinleye1@cardiff.ac.uk).

#### Materials availability

The Horizon Scan Questionnaire is available in the Supplementary Materials of this paper.

### Experimental model and subject details

Ethical approval was granted by Cardiff University, as outlined in the manuscript. All participants in the study provided their informed consent prior to completion of the questionnaire. Participant profile data including gender, age group etc are provided in Table 1.

### Method details

To identify current trends and gaps in marine social sciences, we adapted established horizon scanning methodologies using an online questionnaire to obtain responses from across the global marine science research community. The questionnaire comprised of two sections, each containing a combination of open and closed answer format questions. Questions in section 1 were developed to understand the background of each respondent, particularly in relation to their previous experiences within the marine sciences and their current areas of research. Section 2 then posed a series of open-ended questions to elucidate the respondents’ views on the existing knowledge gaps and future areas of research for the marine social science community. Open-ended questions were used in section 2 of the survey to ensure that responses were not limited in any way (i.e. by discipline, geographic area, scale, etc.) and to ensure that responses were as broad as deemed necessary by the respondents. The questionnaire was piloted through the authors’ professional networks prior to final dissemination. Respondents were recruited using a self-selection, convenience-based sample approach (Bryman, 2012), and criteria for inclusion was provided at the onset of the online survey using the Survey Monkey Platform so that respondents could clearly assess their eligibility to participate. In terms of expected audience, the survey targeted individuals working across the diverse disciplines of the marine social sciences (as explored by McKinley et al., 2020). This included, but was not limited to, marine researchers working within related disciplines of psychology, sociology, anthropology, and political science, among others. By sharing the survey through online networks, including targeted promotion of key groups known to the authors, we sought to facilitate the inclusion of respondents and perspectives from the global south to ensure that results were as broadly representative as possible.

### Quantification and statistical analysis

Due to the inclusion of open and closed questions in the questionnaire, both quantitative and qualitative analysis was carried out on the data. N numbers are quoted alongside the results of both types of analysis within the manuscript.

All qualitative data collected through the online questionnaire were analyzed manually adopting an emergent, inductive coding approach (Glaser and Strauss 1967; Charmaz 2008). First, the data was broadly coded against each of the four research questions outlined in the manuscript. Second, thematic coding of the data was undertaken to develop a coherent set of key themes. This involved the identification and interpretation of patterns or ‘themes’ in the dataset. Emergent categories were developed and revised through a repeated review process to ensure its validity and relevance (Fleming and Vanclay, 2009; Marshall et al., 2011). Content analysis was then used to transform the qualitative data into categorical data. Content analysis quantified the content of the surveys in terms of the pre-determined themes or categories identified through inductive coding (Bryman, 2016). This enabled prioritisation of key themes and further statistical exploration.

For the closed questions, quantitative analysis was carried out in a number of ways. Responses to the survey questions were categorized to themes and collated, and demographic data, such as career stage, location, background and type of scientist was collected for quantitative analysis. A Pearson’s Chi-square statistic was used to assess homogeneity and independence across groups within the sample. This enabled us to explore for any statistically different variations (testing for significance at an alpha value of 0.1 and 0.05; Brereton, 2020). Specifically, we explored if there was a significant difference across the demographic data and trends in marine social science research and the challenges and enablers for marine social science research. While inferences can be made from the survey data alone the quantitative analysis can provide statistical backing to refute any null hypothesis that there is no relationship between any variables. However, it should be noted that this statistical test is highly sensitive to sample size and given the relatively small sample size and in some cases high dimensionality of categorical data (i.e. multiple possible responses for a question) there is a chance that some statistical differences may not be picked up with this test.

## Data Availability

• All data reported in this paper will be shared by the lead contact upon request. • This paper does not report original code. • Any additional information required to reanalyze the data reported in this paper is available from the lead contact upon request.
