# Supplementary material for: Development and expansion in the marine social sciences: Insights from the global community
Source: iScience. 2022 Jul 11;25(8):104735. doi: 10.1016/j.isci.2022.104735 (PMC9356031; doi:10.1016/j.isci.2022.104735)
Supplement: Document S1. Tables S1–S6 [file mmc1.pdf]

iScience, Volume *25*

Supplemental information

Development and expansion in the marine social sciences: Insights from the global community

Emma McKinley, Rachel Kelly, Mary Mackay, Rebecca Shellock, Christopher Cvitanovic, and Ingrid van Putten

Supplementary Material

Table SM1: Respondent Career Stage and type of science, related to Table 1.

Career stage	A natural scientist	A social scientist	Both
Undergraduate student	0	1	1
Postgraduate student (Masters)	2	4	7
Postgraduate student (PhD)	3	13	15
Early – up to 5 years post PhD	6	6	10
Mid – 6 – 20 years post PhD	13	6	0
Late – over 20 years	4	4	2
Other	2	4	2

Table SM2: Respondent location and type of science, related to Table 1.

Row Labels	A natural scientist	A social scientist	Both
Africa	0	2	3
Asia	1	1	4
Australia/ NZ	6	4	4
Central & South America	3	1	3
Europe	15	17	12
North America	5	13	11

Table SM3. Proportion of research priorities for each science type, related to Table 5.

	Community involvement & ocean literacy	Research Landscape	Communication & capacity building	Governance & decision-making processes	Marine Sectors and Research Areas	Total (Number)
Social	0.36	0.17	0.11	0.19	0.17	108
Natural	0.29	0.19	0.06	0.34	0.11	79
Both	0.30	0.16	0.08	0.29	0.17	145

Table SM4: Proportion of research priorities for each career stage, related to Table 5.

**Career stage**	Community involvement & ocean literacy	Research Landscape	Communication & capacity building	Governance & decision-making processes	Marine Sectors and Research Areas	Total (Number)
Early – up to 5 years post PhD	0.32	0.23	0.07	0.21	0.17	75
Mid – 6 – 20 years post PhD	0.29	0.25	0.07	0.26	0.14	73
Late – over 20 years	0.32	0.06	0.09	0.26	0.26	34
student	0.33	0.11	0.10	0.33	0.13	132
Other	0.28	0.22	0.17	0.17	0.17	18

Table SM5: Proportion of research priorities for each employment status, related to Table 5.

**Employment Status**	Community involvement & ocean literacy	Research Landscape	Communication & capacity building	Governance & decision-making processes	Marine Sectors and Research Areas	Total (N)
fixed term	0.30	0.23	0.11	0.25	0.10	79
permanent	0.34	0.15	0.07	0.24	0.20	143
In education	0.30	0.15	0.09	0.32	0.14	79
not in workforce	0.33	0.05	0.05	0.43	0.14	21
Unknown	0.10	0.40	0.20	0.20	0.10	10

Table SM6: Proportion of research priorities for each employment status, related to Table 5.

Continent	Community involvement & ocean literacy	Research Landscape	Communication & capacity building	Governance & decision-making processes	Marine Sectors and Research Areas	Total (N)
Africa	0.50	0.05	0.14	0.23	0.09	22
Asia	0.25	0.20	0.15	0.20	0.20	20
Australia	0.27	0.10	0.07	0.30	0.27	30
Central & south America	0.39	0.00	0.17	0.39	0.06	18
Europe	0.31	0.19	0.07	0.30	0.13	150
North America	0.29	0.22	0.09	0.22	0.18	92
